# Soil microbial biomass in tropical coastal shelterbelts of Hainan Island: seasonal characteristics and responses to environmental factors

**DOI:** 10.3389/fpls.2026.1925888

**Published:** 2026-08-26

**Authors:** Haihui Chen, Zhipan Lin, Leyu Tian, Yiqing Chen, Zongzhu Chen, Juzhi Liang, Xiangling Lei, Shaofeng Su, Shouqian Nong

**Affiliations:** 1 Hainan Academy of Forestry (Hainan Academy of Mangrove), Haikou, China; 2 Hainan Wenchang Forest Ecosystem Observation and Research Station, Wenchang, China; 3 Key Laboratory of Tropical Forestry Resources Monitoring and Application of Hainan Province, Haikou, China; 4 The Innovation Platform for Academicians of Hainan Province, Haikou, China

**Keywords:** environmental drivers, microbial entropy, seasonal dynamics, soil microbial biomass, tropical coastal shelterbelt

## Abstract

**Introduction:**

Soil microorganisms regulate plant nutrition through nutrient transformation, and forest soil microbial biomass is crucial for ecosystem health and nutrient cycling. Tropical coastal shelterbelts are essential for coastal ecological security, but seasonal microbial biomass variation and environmental controls in forests remain insufficiently understood.

**Methods:**

To determine how soil microbial biomass changes seasonally in tropical coastal shelterbelts and which environmental factors regulate this variation, four representative shelterbelt types in Wenchang, Hainan, were examined: secondary forest (SF), mixed forest, *Cocos nucifera* forest, and *Casuarina equisetifolia* forest. The differences in soil microbial biomass characteristics between wet and dry seasons were compared. Correlation analysis and redundancy analysis (RDA) were adopted as core analytical approaches to identify associations between environmental factors and microbial biomass. Exploratory structural equation modeling was performed as supplementary analysis to visualize potential regulatory pathways.

**Results:**

During both seasons, SF exhibited significantly higher microbial biomass phosphorus, microbial biomass nitrogen, and microbial biomass carbon than the other forest types. From the dry to wet season, microbial entropy, microbial biomass stoichiometric ratios, and microbial biomass levels increased across all forest stands. The direction of the associations between microbial biomass, its stoichiometric ratios, and environmental factors was generally consistent between seasons, whereas the strength of these associations varied seasonally. RDA identified soil total phosphorus, available phosphorus (AP), and precipitation (PRCP) as the main factors associated with variations in microbial biomass levels and their elemental stoichiometric ratios. Their dominant roles were not altered by seasonal transition. Exploratory supplementary analysis further supported the seasonal reconfiguration of environmental regulatory pathways.

**Discussion:**

These findings demonstrate the stand- and season-specific patterns of soil microbial biomass in tropical coastal shelterbelts and clarify how the responses of microbial biomass to environmental factors are reshaped by seasonal precipitation variations.

## 1 Introduction

Within forest ecosystems, soil microbial biomass is the metabolically active core of soil biota and drives key belowground ecological processes, including litter decomposition, nutrient release, and organic matter transformation (Cui et al., 2021; Xu et al., 2013). By mediating the mineralization and sequestration of carbon, nitrogen, and phosphorus, microbial biomass directly regulates soil nutrient availability and turnover. These processes, in turn, influence plant nutrient uptake and forest stand productivity (Camenzind et al., 2018; Spohn, 2016). Plants shape soil microbial biomass and community structure through litter inputs and root exudates, forming a tightly coupled plant–soil–microbe system that drives nutrient cycling and plant adaptation to stressful habitats (Feng et al., 2022; Chen et al., 2024b).

Soil microbial biomass is defined as the collective mass of microorganisms smaller than 5 × 10^3^ μm^3^ that occur outside living plant tissues. It mainly includes microbial biomass phosphorus (MBP), microbial biomass nitrogen (MBN), and microbial biomass carbon (MBC), and is widely used as the metric for measuring soil microbial activity (Wu et al., 2025). Soil microbial biomass is associated with phosphorus, nitrogen, and soil carbon cycling (Chen et al., 2024a; Spohn, 2016; Cui et al., 2025). By contributing to carbon cycling and nutrient turnover, it supports soil function and strengthens ecosystem buffering capacity after disturbance (Xu et al., 2013). Stoichiometric ratios can indicate soil nutrient constraints and forest nutrient demand (Asada et al., 2022; Cui et al., 2021; Rosinger et al., 2022). Microbial entropy reflects the efficiency with which soil microorganisms use available resources and provides a useful proxy for predicting changes in soil nutrient pools and evaluating soil degradation (Ding et al., 2025). Therefore, a systematic assessment of forest soil microbial biomass is important for identifying regional soil nutrient limitations, improving our understanding of nutrient cycling in forest ecosystems, and supporting sustainable forest management and ecological function maintenance.

Climatic conditions, biological factors, and soil physicochemical properties regulate soil microbial biomass (Thakur et al., 2015; Wang et al., 2023; Xue et al., 2019). Soil microbial biomass is highly sensitive to seasonal variations (Wang et al., 2016). Climatic factors, such as temperature and precipitation, alter soil hydrothermal conditions, directly affecting microbial metabolism and population turnover (Chen et al., 2025b). Meanwhile, soil pH, moisture, and nutrient availability determine the quantity and form of carbon, nitrogen, and phosphorus substrates available to microorganisms (Dębska et al., 2024). These environmental factors interact jointly to shape microbial habitat suitability. Numerous studies have examined soil microbial biomass patterns across land-use regimes, forest types, and seasonal gradients (Cai et al., 2018; Jiang et al., 2021; Wang et al., 2016, 2023; Zhang et al., 2018; Dębska et al., 2024). Tropical forest ecosystems have also received substantial attention (Camenzind et al., 2018; Chen et al., 2020; Dietterich et al., 2022). However, tropical studies have primarily focused on inland primary rainforests and secondary forests. Research on tropical coastal shelterbelts remains limited, and neither seasonal microbial biomass dynamics nor their environmental drivers have been systematically characterized in this distinctive coastal sandy habitat.

Tropical coastal shelterbelts provide vital ecological functions, including windbreak and sand fixation, soil nutrient conservation, and regional climate regulation. At the land-sea interface, these stands experience persistent environmental stress from sandy soils, low nutrient availability, monsoons, salt spray, and typhoons (Chen et al., 2025a; Yuan et al., 2021). Unlike inland forests with relatively stable soil substrates, coastal sandy habitats have poor water retention, intense nutrient leaching, and intermittent salt stress. These conditions impose unique selective pressures and produce distinct microbial biomass responses to environmental fluctuations (Chen et al., 2019; Ni et al., 2022; Yuan et al., 2021). Consequently, they are more ecologically vulnerable and sensitive than most inland forest ecosystems (Naren and Maity, 2024; Xu et al., 2023; Nong et al., 2025). Distinct climatic conditions and nutrient-poor soils may strongly constrain soil microorganisms and shape region-specific controls on microbial biomass. Nevertheless, existing coastal forest studies have focused mainly on vegetation restoration, soil respiration dynamics, and plant nutrient stoichiometry. Systematic investigations of the seasonal drivers of soil microbial biomass in this shelterbelt habitat remain scarce (Chen et al., 2025a; Nong et al., 2025; Yuan et al., 2021). The responses of soil microbial biomass to seasonal environmental changes and their underlying drivers in tropical coastal shelterbelts remain unclear. Therefore, further research is needed to clarify microbial adaptation under habitat-specific constraints and support the maintenance and management of shelterbelt ecological functions.

To address these research gaps, representative tropical coastal shelterbelt forests in Wenchang City, Hainan Province were selected as the study system. This study addressed two core scientific questions. (1) What are the characteristics of soil microbial biomass across different forest types and seasons in tropical coastal shelterbelts? (2) What are the key environmental drivers influencing soil microbial biomass and its seasonal dynamics? Four representative stand types, including secondary forest (SF), mixed forest (MF), *Cocos nucifera* forest (CO), and *Casuarina equisetifolia* forest (CA), were used to characterize soil microbial biomass patterns and identify their key environmental regulators across seasons. These findings reveal microbial adaptation strategies to coastal sandy habitats, advance the understanding of microbial ecology in tropical coastal shelterbelts, and provide scientific support for sustainable shelterbelt management.

## 2 Materials and methods

### 2.1 Overview of the study area

The research site was located in Wenchang City, Hainan Province, within the geographical range of 19°21′N–20°01′N and 110°28′E–111°03′E. The area has a typical tropical maritime monsoon climate with a mean annual temperature of approximately 23.9 °C. Seasonal temperature variation is limited, and the coolest period occurs from December to February. Annual precipitation exceeds 1,600 mm and shows distinct alternation between dry and wet seasons. The rainy season covers the period between May and October and accounts for more than 80% of the annual rainfall, whereas the dry season covers the months between November and April of the next year. A low-lying coastal plain terrace with an average elevation of 42.6 m was selected as the study area. As the main soil type, the sandy loam is derived from coastal sedimentary parent material, which is acidic and has relatively low nutrient levels (Chen et al., 2025a).

### 2.2 Plot setup

**Figure 1:**
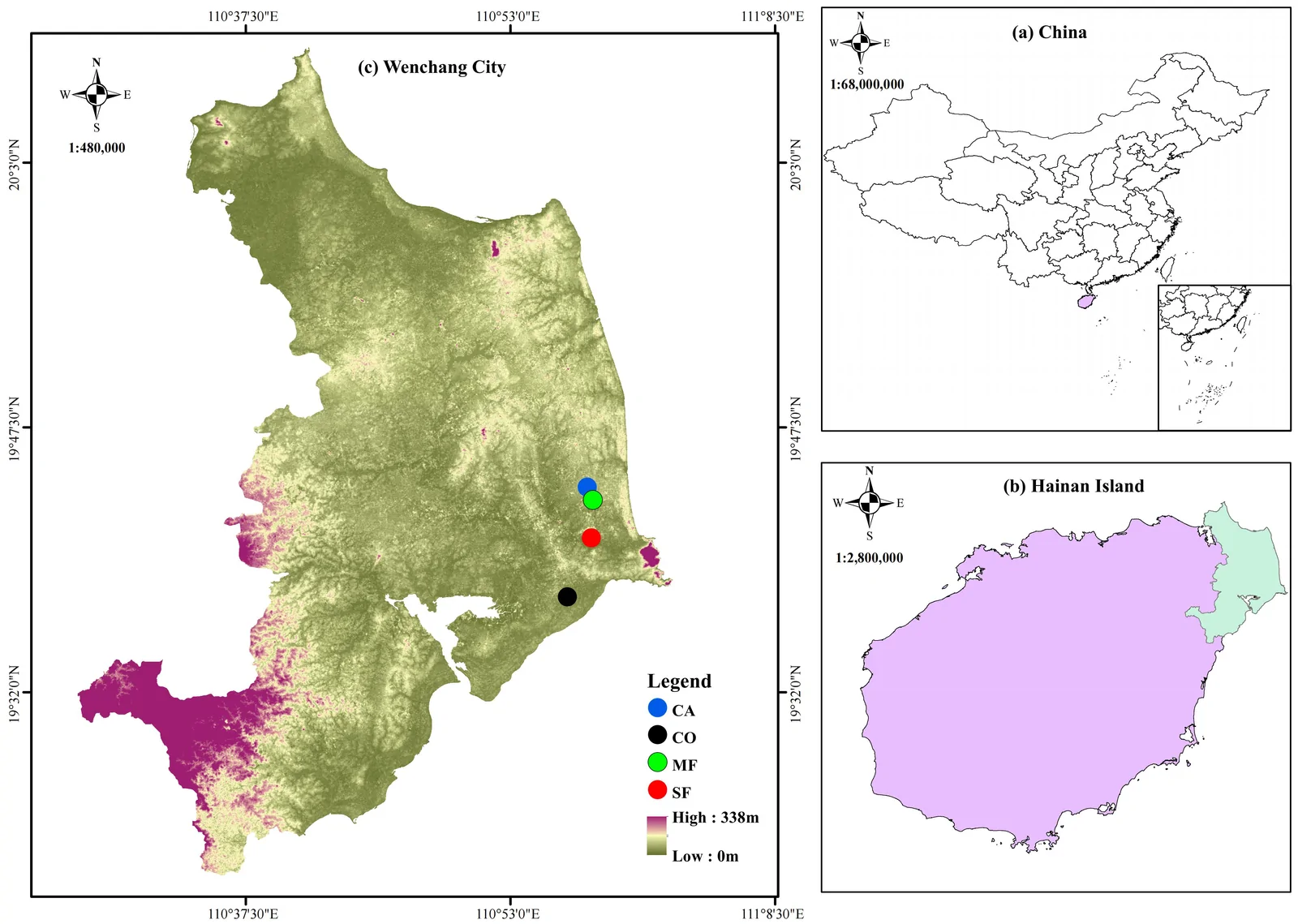
Map of the study area and distribution of sampling plots. CA, CO, MF, and SF denote *C. equisetifolia* forest, *C. nucifera* forest, mixed forest, and secondary forest, respectively. The same abbreviations are used in **Figures 2**–**6**. **(a)** China; **(b)** Hainan Island; **(c)** Wenchang City.

This study was conducted at the Wenchang Forest Ecosystem Long-Term Observation Station in Hainan. The adopted forest stand types involved SF, MF, CO, and CA. For each stand type, three survey plots (20 m × 20 m) with a minimum 20-meter spacing were established. The geographic locations are illustrated in **Figure 1**, and the basic properties of each type are listed in **Table 1**.

**Table 1:** Characteristics of four tropical coastal shelter forest stands.

Index	Stand			
	CA	CO	MF	SF
Soil type	Sandy loam	Sandy loam	Sandy loam	Sandy loam
Age of the forest (years)	10	10	10	10
Major tree species composition	*Casuarina equisetifolia*	*Cocos nucifera*	*Pinus caribaea;* *Casuarina equisetifolia;* *Eucalyptus tereticornis;* *Acacia auriculiformis*	*Castanopsis wenchangensis;* *Heptapleurum heptaphyllum;* *Calophyllum inophyllum;Litsea pseudoelongata;* *Symplocos poilanei;*
Mean tree height (m)	10.1 ± 0.3	9.9 ± 0.3	11.6 ± 2.8	9.3 ± 3.7
Mean diameter at breast height (cm)	18.0 ± 0.4	24.2 ± 1.5	19.6 ± 3.5	18.0 ± 5.3

CA, CO, MF, and SF denote *C. equisetifolia* forest, *C. nucifera forest*, mixed forest, and secondary forest, respectively. The same abbreviations are used in **Table 2**.

### 2.3 Determination of environmental factors

#### 2.3.1 Hydrological and thermal factors

Automatic weather stations (Model: FIM. MEM09, Beijing Global Forest Technology Co., Ltd., China) were implemented in each forest stand for continuously monitoring the main hydrological and thermal parameters of the forest ecosystems. The monitored variables included soil water content (SW), soil temperature (ST), relative humidity (RH), air temperature (TEMP), and precipitation (PRCP), allowing the real-time dynamic recording of these parameters. The monthly averages of each hydrothermal factor were calculated for both the dry and wet seasons of November 2023 to April 2024 and May 2024 to October 2024, respectively. In subsequent analyses, all hydrothermal indicators were represented by their monthly averages during both seasons.

#### 2.3.2 Soil chemical parameters

Soil samples were collected in the dry season (December 2023) and the wet season (July 2024). A five-point sampling scheme was used, with sampling points arranged at the four corners and center of each plot. Surface soil samples from the 0–10 cm layer were collected using a 5-cm-diameter soil auger. After collection, plant roots and stone fragments were manually removed. The samples were homogenized, divided into two subsamples, placed in sterile sealed bags, and labeled. They were transported to the laboratory under low-temperature conditions for further analysis. Both subsamples were adopted for the determination of soil microbial biomass and soil chemical analysis.

For chemical analysis, the samples were air-dried and processed through a 100-mesh sieve. Soil pH was assessed using the potentiometric method at a 1:2.5 soil-to-water ratio. Total carbon (TC) was measured using potassium dichromate oxidation with external heating. Total nitrogen (TN), total phosphorus (TP), available nitrogen (AN), and available phosphorus (AP) were determined using the semi-micro Kjeldahl method, the acid-soluble molybdenum-antimony colorimetric method, and a flow injection analyzer after extraction with KCl solution, and the sodium bicarbonate extraction-molybdenum-antimony colorimetric method (Tan et al., 2020), respectively.

### 2.4 Determination of soil microbial biomass

MBC and MBN were determined using the chloroform fumigation-extraction method. Briefly, fresh soil samples were fumigated with ethanol-free chloroform for 24 h before extraction with 0.5 mol L^−1^ K_2_SO_4_. Extractable organic C and N concentrations were measured using a Multi N/C 3000 analyzer (Analytik Jena, Germany). MBP was measured using chloroform fumigation, followed by extraction with 0.5 mol L^−1^ NaHCO_3_ (pH 8.5). The conversion factors *K_C_*, *K_N_*, and *K_P_* were 0.45 (Vance et al., 1987), 0.54 (Brookes et al., 1985), and 0.40 (Brookes et al., 1982), respectively. Microbial biomass stoichiometry was determined using mass-based MBC/MBN, MBC/MBP, and MBN/MBP ratios.

Microbial entropy indices (qMBC, qMBN, and qMBP) were calculated using Equations 1–3 (Anderson and Domsch, 1989):

(1) qMBC(%)=MBC/TC×100

(2) qMBN(%)=MBN/TN×100

(3) qMBP(%)=MBP/TP×100

These indices represent the proportion of total soil C, N, and P stored in the microbial biomass. They are commonly used as sensitive indicators of soil nutrient availability and microbial resource use efficiency.

### 2.5 Data processing and analysis

Preliminary data processing was performed using Excel 2024. The variations in soil chemical properties and soil microbial biomass characteristics among precipitation seasons and forest stand types were tested using one-way ANOVA in SPSS 27, with significance set at α = 0.05. When significant differences were detected, the LSD multiple comparison test was applied. Spearman’s rank correlation analysis was conducted in R version 4.5.1 using the Hmisc package to analyze the correlation between environmental factors and soil microbial biomass. Redundancy analysis (RDA) was performed using the vegan package to assess the correlations among environmental factors, soil microbial biomass levels, and microbial biomass stoichiometric ratios. To simplify response variables for subsequent path analysis, principal component analysis (PCA) was performed to extract PC1 scores for microbial biomass levels and stoichiometric ratios respectively, which served as composite indicators for model construction. On this basis, a piecewise structural equation model was constructed using the piecewiseSEM package as exploratory path analysis to visualize potential direct and indirect regulatory pathways of environmental factors on microbial biomass. Due to the limited sample size, this analysis is presented in the Appendix as supplementary exploratory material and is not regarded as a core confirmatory result.

## 3 Results and analysis

### 3.1 Environmental characteristics of tropical coastal shelterbelts

#### 3.1.1 Hydrological and thermal characteristics

As shown in **Table 2**, the seasonal patterns of hydrological and thermal factors were highly consistent across the four forest stands. The dry-wet seasonal shift was characterized by increases in TEMP, RH, PRCP, ST, and SW, reflecting the distinct dry–wet seasonal alternation associated with the tropical monsoon climate of the study area. Although the hydrological and thermal conditions differed among stand types, the overall patterns were broadly similar. In both seasons, SF showed the lowest TEMP and ST but the highest RH and SW among all stands. Moreover, the hydrological and thermal factors exhibited relatively small seasonal fluctuations, indicating a higher seasonal stability of the understory hydrothermal environment. In contrast, CO showed the largest seasonal changes in PRCP and ST, suggesting stronger fluctuations in the hydrothermal environment.

**Table 2:** Hydrological and thermal characteristics of different forest types.

Hydrological factors	Season	Stand type			
		CA	CO	MF	SF
TEMP (°C)	Dry season	22.85	22.90	22.77	22.28
	Wet season	27.65	27.28	27.27	26.87
RH (%)	Dry season	80.48	79.78	80.73	82.47
	Wet season	82.95	81.50	83.43	85.32
PRCP (mm)	Dry season	39.65	28.39	35.53	28.83
	Wet season	244.65	283.26	255.08	254.56
ST (°C)	Dry season	23.05	23.23	23.53	21.13
	Wet season	28.68	30.33	29.81	26.25
SW (%)	Dry season	6.80	11.71	7.31	23.21
	Wet season	8.76	16.43	8.25	28.38

SW, ST, RH, TEMP, and PRCP refer to the monthly metrics of average soil moisture content, average soil temperature, average relative humidity, average air temperature, and average precipitation, respectively.

#### 3.1.2 Soil chemical characteristics

**Figure 2:**
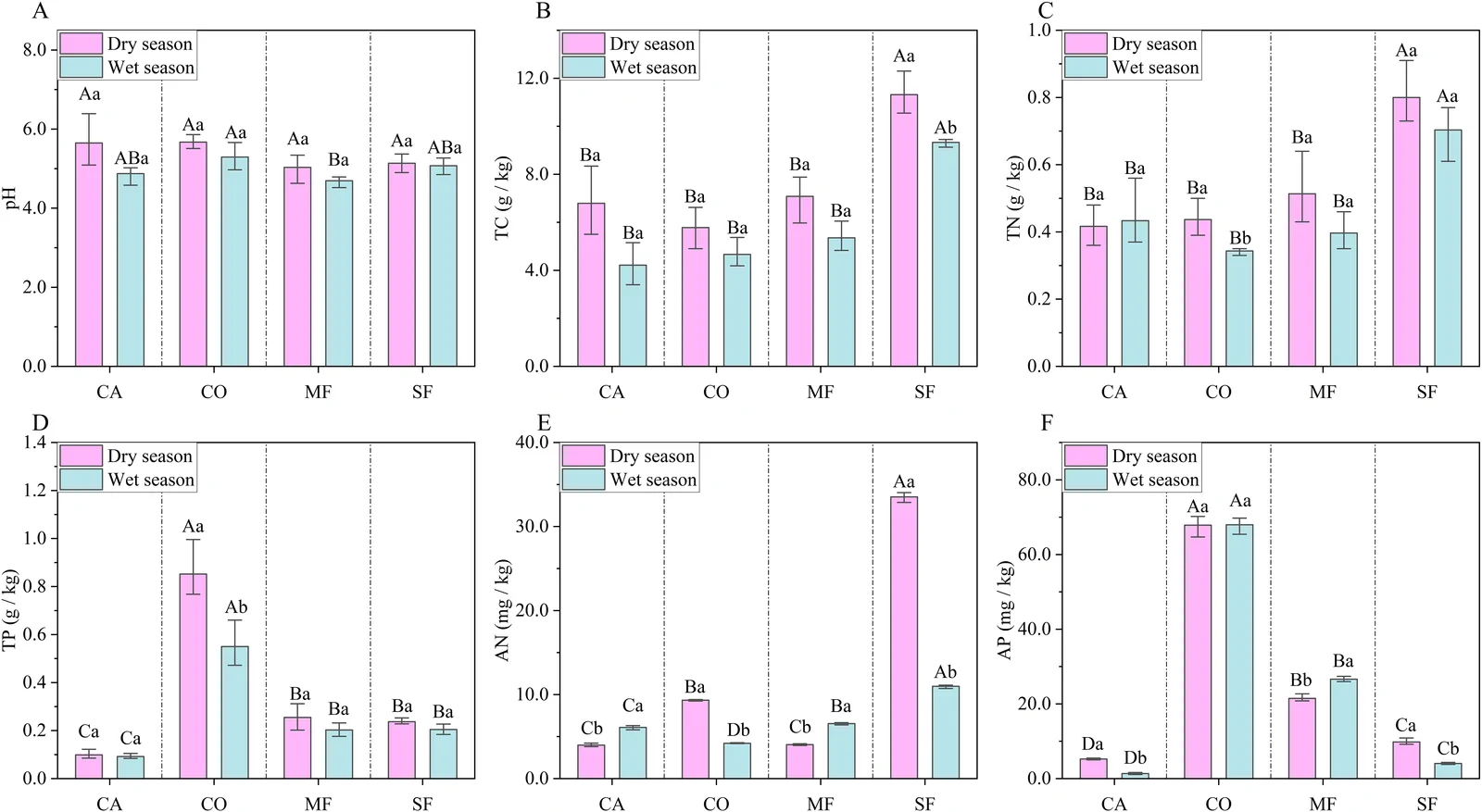
Soil chemical characteristics of forest types. TP, TN, and TC denote the contents of total soil phosphorus, total soil nitrogen, and soil organic carbon, respectively. Lowercase letters represent significant differences (*P*< 0.05) among precipitation seasons in the same forest type, and uppercase letters represent significant differences (*P*< 0.05) among forest types in the same precipitation season. Same in **Figures 3** and **4**. **(A)** pH; **(B)** TC; **(C)** TN; **(D)** TP; **(E)** AN; **(F)** AP.

As shown in **Figure 2**, the soil in the study area was generally acidic, and the soil nutrient content differed among the four forest stands. SF had the highest carbon and nitrogen nutrient levels, with TC, TN, and AN being significantly higher than those in the other stands during both dry and wet seasons (*P*< 0.05). CO showed a distinct advantage in phosphorus availability, as its TP and AP contents were significantly higher than those of the other stands in both seasons (*P*< 0.05). After the transition from the dry season to the wet season, pH, TC, TN, and TP decreased across all stands. Except for MF, AN and AP showed decreasing trends in the remaining stands.

### 3.2 Soil microbial biomass characteristics in tropical coastal shelterbelts

**Figure 3:**
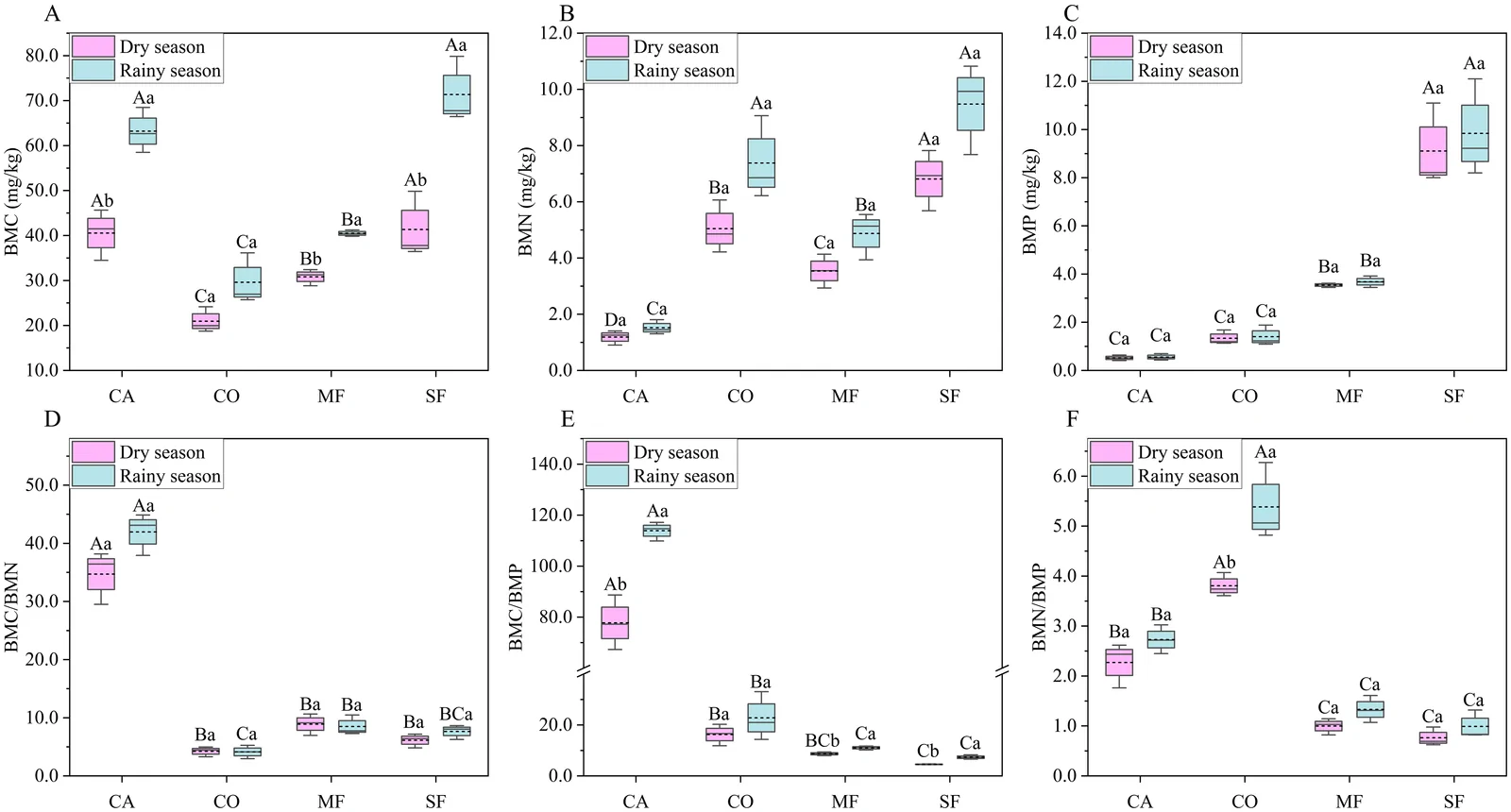
Soil microbial biomass characteristics of different forest types. MBP, MBN, and MBC denote soil microbial biomass phosphorus, soil microbial biomass nitrogen, and soil microbial biomass carbon, respectively. Same in **Figures 5** and **6**. **(A)** BMC; **(B)** BMN; **(C)** BMP; **(D)** BMC/BMN; **(E)** BMC/BMP; **(F)** BMN/BMP.

**Figure 3** illustrates the evident differences in microbial biomass phosphorus, nitrogen, and carbon and their stoichiometric ratios among the forest stands. SF had the greatest accumulation of MBP, MBN, and MBC, with the highest values for all three microbial biomass components in both seasons. In contrast, CA and CO exhibited distinct patterns in microbial biomass stoichiometry. CA demonstrated significantly higher MBC/MBP and MBC/MBN ratios than other forest types during both seasons (*P*< 0.05), whereas CO exhibited a substantially higher MBN/MBP ratio than other stands during both seasons (*P*< 0.05). Throughout the selected period, MBP, MBN, MBC, and stoichiometric ratios increased in all four forest types.

**Figure 4:**
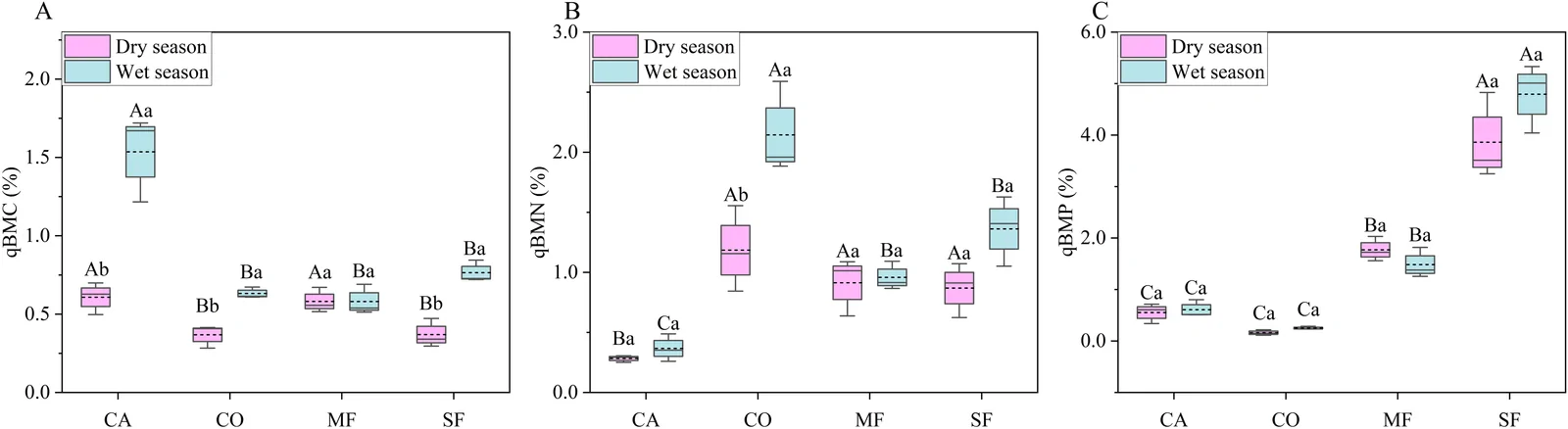
Soil microbial entropy characteristics of different forest types. qMBP, qMBN, and qMBC denote soil microbial entropy phosphorus, soil microbial entropy nitrogen, and soil microbial entropy carbon, respectively. **(A)** qBMC; **(B)** qBMN; **(C)** qBMP.

**Figure 4** shows the significant variations in soil microbial entropy among forest types. Throughout the testing period, the highest qMBP, qMBN, and qMBC values were observed in SF, CO, and CA, respectively. During the wet season, CA, CO, and SF exhibited significantly higher corresponding indicators than the other stands (*P*< 0.05). With seasonal transition, soil microbial entropy increased across all four forest types. This increase was particularly evident for qMBC, which significantly increased in all stands except MF (*P*< 0.05).

### 3.3 Relationship between soil microbial biomass and environmental factors in tropical coastal shelterbelts

#### 3.3.1 Correlations between environmental factors and soil microbial biomass

**Figure 5:**
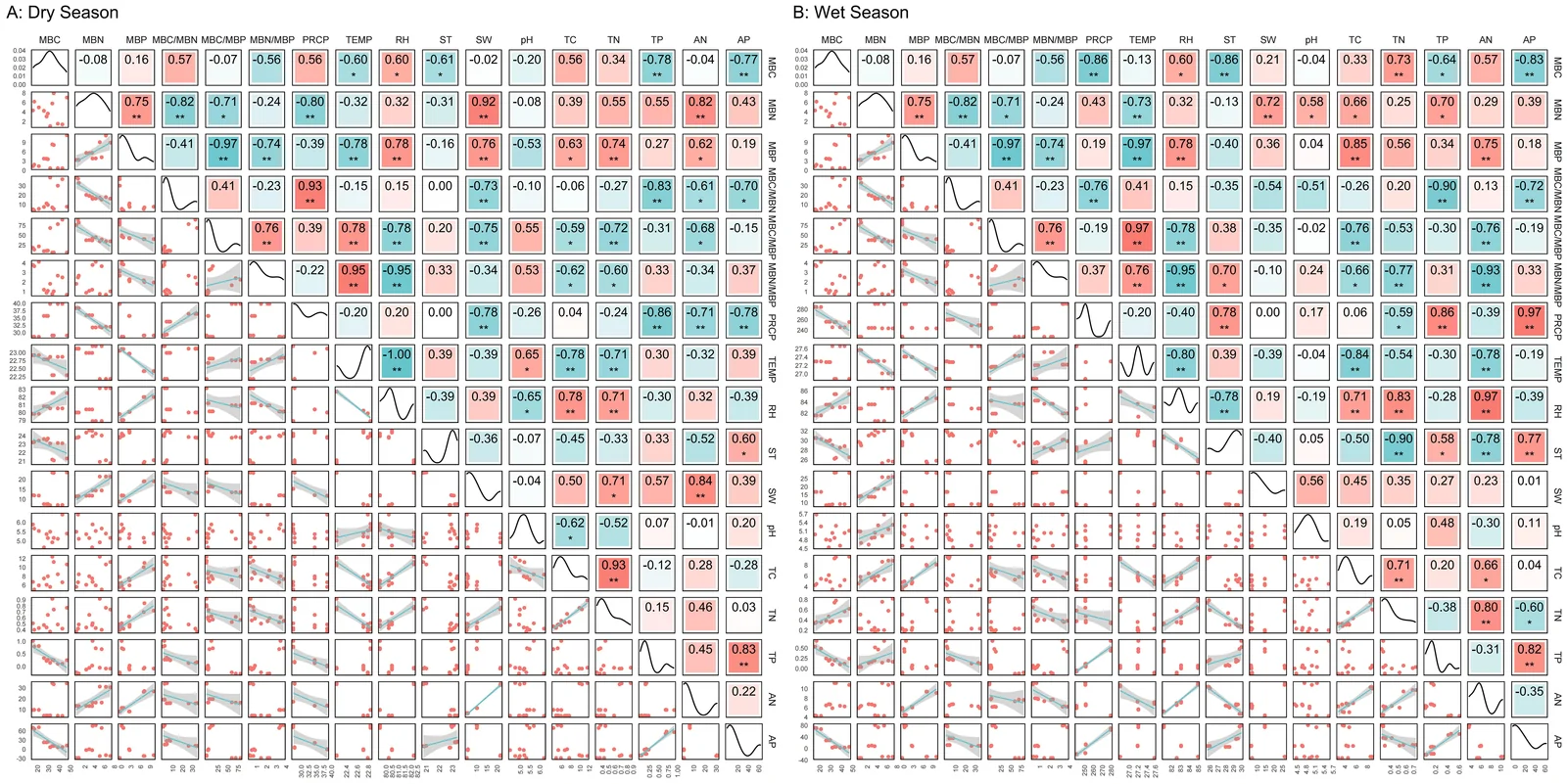
Correlation analysis of soil microbial biomass, stoichiometric ratios, and environmental factors. *P< 0.05, **P< 0.01. MBP, MBN, and MBC denote soil microbial biomass phosphorus, soil microbial biomass nitrogen, and soil microbial biomass carbon, respectively. AP, AN, TP, TN, TC, pH, SW, ST, RH, TEMP, and PRCP represent soil available phosphorus content, soil available nitrogen content, soil total phosphorus content, soil total nitrogen content, soil organic carbon content, soil pH, monthly average soil water content, monthly average soil temperature, monthly average air humidity, monthly average precipitation, and monthly average air temperature, respectively. Same in **Figure 6**. **(A)** Dry Season; **(B)** Wet Season.

As shown in **Figure 5**, the associations of soil microbial biomass (MBP, MBN, and MBC) and its stoichiometric ratios (MBN/MBP, MBC/MBP, and MBC/MBN) with environmental factors differed to some extent between seasons. During the dry season, the soil microbial biomass exhibited a negative correlation with TEMP, ST, and soil phosphorus variables (TP and AP) but was positively correlated with RH, SW, TC, and soil nitrogen variables (TN and AN). In contrast, the microbial biomass stoichiometric ratios were positively correlated with TEMP and negatively correlated with RH, SW, TC, and soil nitrogen variables. After the seasonal transition, the strength of these correlations changed markedly. The negative correlations of soil microbial biomass with TEMP and ST became stronger, whereas those with soil phosphorus variables (TP and AP) weakened. The positive correlations with RH and SW weakened, whereas the positive correlation with TC became stronger. Positive correlations with soil nitrogen variables (TN and AN) remained strong. Among the stoichiometric ratios, the relationship between MBC/MBN and PRCP shifted from a significant positive correlation to a significant negative correlation (*P*< 0.01), and the negative correlations between each ratio and nutrient variables generally intensified.

These results indicate that seasonal variation primarily alters the strength rather than the overall direction of the associations between soil microbial biomass, its stoichiometric ratios, and environmental factors. During the dry season, microbial characteristics were more closely associated with temperature, moisture, and phosphorus. During the wet season, the associations with precipitation and soil carbon and nitrogen nutrients became more pronounced.

#### 3.3.2 RDA of environmental factors and soil microbial biomass

**Figure 6:**
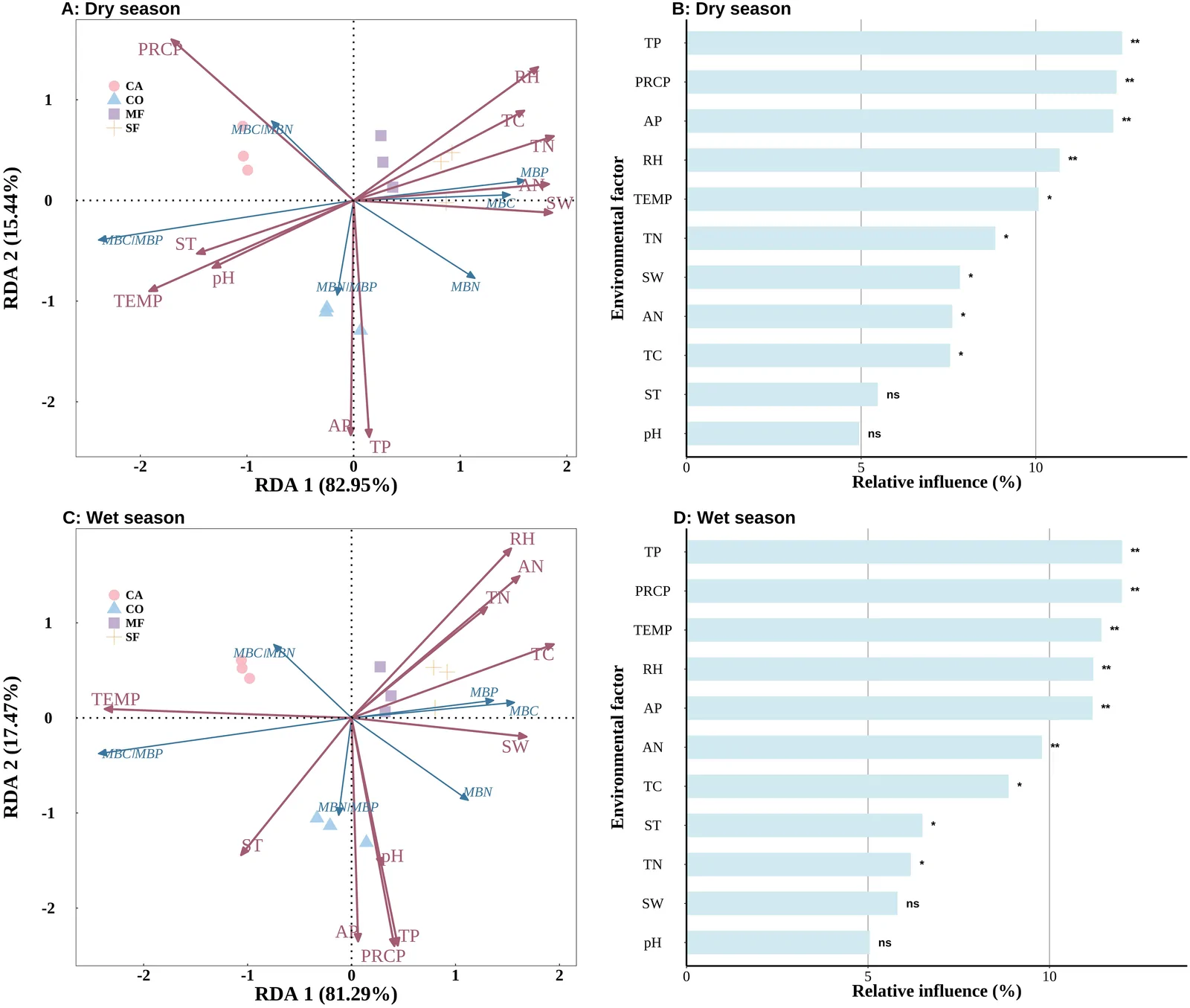
RDA of environmental factors, stoichiometric ratios, and soil microbial biomass levels during both seasons. **P< 0.01, *P< 0.05, ns: *P* > 0.05. **(A)** RDA ordination in dry season; **(B)** Relative influence of environmental factors in dry season; **(C)** RDA ordination in wet season; **(D)** Relative influence of environmental factors in wet season.

RDA showed that during the dry season, the soil microbial biomass was positively correlated with RH, TC, TN, AN, and SW but negatively associated with ST, TEMP, and pH. The stoichiometric ratios of soil microbial biomass showed an opposite association pattern with these environmental factors. The first two RDA axes explained 98.39% of the total variance in the soil microbial biomass and its stoichiometric ratios. Among the environmental variables, TP, PRCP, and AP were the main explanatory factors, accounting for 12.47%, 12.31%, and 12.22% of the total variance, respectively. RH, TEMP, and TN contributed 10.68%, 10.08%, and 8.84%, respectively, whereas pH and ST had relatively weaker explanatory effects (**Figures 6A, B**).

During the wet season, soil microbial biomass was positively associated with AN, TN, TC, and SW but negatively associated with TEMP and ST. Microbial biomass stoichiometric ratios showed an opposite association pattern. The first two RDA axes explained 98.76% of the total variance. TP and PRCP were the main explanatory factors, accounting for 12.00% and 11.99% of the variance, respectively, followed by TEMP, RH, and AP, which explained 11.43%, 11.20%, and 11.18%, respectively. The explanatory contributions of pH and SW were relatively low (**Figures 6C,D**).

These results suggest that the association patterns among soil microbial biomass, its stoichiometric ratios, and environmental factors were largely consistent between both seasons. Soil phosphorus components and precipitation were the main factors associated with variations in microbial characteristics. Seasonal changes did not alter the dominant roles of these environmental factors but only led to minor shifts in their explanatory contributions.

To further explore the potential direct and indirect regulatory pathways and their seasonal reconfiguration, we conducted piecewise structural equation modeling as exploratory path analysis. Constrained by the limited sample size, this analysis is only used for exploratory interpretation rather than core confirmatory conclusions, and its results are generally consistent with the above correlation and RDA findings. Detailed model parameters and pathway diagrams are provided in the Appendix (**Supplementary Figure S1**).

## 4 Discussion

### 4.1 Environmental characteristics of tropical coastal shelterbelts

Seasonal variations in precipitation markedly altered the environmental conditions of tropical coastal shelterbelts, with differences observed among forest stands. The results showed highly consistent seasonal patterns in hydrothermal factors. Throughout the testing seasons, TEMP, RH, PRCP, ST, and SW increased simultaneously, reflecting the distinct dry–wet seasonal alternation in the study area. At the stand level, SF exhibited the lowest TEMP and ST, the highest RH and SW, and the smallest seasonal fluctuations. This pattern may be mainly associated with the complex community structure, high canopy closure, and abundant litter accumulation of SF stands, which can regulate the understory microenvironment, buffer seasonal fluctuations in hydrothermal factors, and improve environmental stability. This is consistent with the findings of Hardwick et al. (2015) and suggests that complex stand structures can buffer variations in hydrothermal conditions. In contrast, CO showed the highest seasonal variations in PRCP and ST, indicating the strongest hydrothermal fluctuations. This pattern may be related to the relatively sparse canopy structure, low litter cover, and weaker capacity for microenvironmental regulation (Pallavi et al., 2023).

Regarding soil chemical properties, SF had significantly higher TC, TN, and AN contents than the other stands, which was closely associated with the differences in nutrient accumulation capacity among stand types. As a natural secondary forest, SF can be characterized by high community diversity, substantial litter input, and slow decomposition, allowing effective accumulation of N and C nutrients (Zhu et al., 2022). Luo et al. (2025) reported that the natural secondary forests in Hainan had higher-quality litter and stronger nutrient supply capacity than rubber plantations, thereby promoting the accumulation of soil organic N and C. Nutrient content and soil pH decreased in all forest types. This pattern is consistent with the results of Chen et al. (2019) for subtropical forests. This decline may be primarily explained by concentrated and intense rainfall during the wet season, high permeability of coastal sandy loam, and severe nutrient leaching. In addition, enhanced microbial activity during the wet season may increase soil nutrient consumption (Sun et al., 2024), contributing to an overall decrease in soil nutrient content. These seasonal changes provide an important basis for subsequent analyses of the mechanisms driving microbial biomass dynamics.

### 4.2 Variations in soil microbial biomass characteristics in tropical coastal shelterbelts

The present study showed that the soil microbial biomass, microbial biomass stoichiometric ratios, and microbial entropy in tropical coastal shelterbelts increased from the dry season to the wet season. This pattern is consistent with observations reported for tropical forests in previous studies (Dietterich et al., 2022; Fujii et al., 2024; Singh et al., 2025). This increase was closely associated with improved hydrothermal conditions during the wet season. In the present study, TEMP, RH, PRCP, ST, and SW all increased during the seasonal transition from dry to wet conditions. These conditions are favorable for enhancing microbial metabolic activity and accelerating soil nutrient transformation, thereby increasing nutrient availability for microorganisms and improving microbial nutrient utilization efficiency (Singh et al., 2025).

In both seasons, SF consistently had higher MBC, MBN, and MBP levels than the other stand types. This agrees with evidence that natural secondary forests support more microbial biomass than planted forests (Yang et al., 2010). Notably, the microbial biomass difference between secondary forests and pure plantations is greater in this coastal sandy habitat than in most inland forest ecosystems. Harsh edaphic conditions (coarse texture, low nutrient retention, and intermittent salt stress) amplify the buffering effect of complex stand structures on soil microbial habitats. The higher microbial biomass in SF may be mainly attributed to its stable hydrothermal conditions and abundant soil carbon and nitrogen nutrients, which provide favorable energy and nutrient conditions for microbial growth and reproduction. Feng et al. (2022) showed that increased plant inputs, including litter and root exudates, could substantially increase soil microbial biomass. As a natural secondary forest, SF has high community diversity and abundant litter input, which can provide rich substrates for microbial growth. In addition, higher tree species diversity may promote microbial biomass accumulation by enhancing microbial carbon use efficiency and improving substrate availability (Duan et al., 2023). In turn, greater microbial biomass accelerates soil nutrient mineralization and enhances plant nutrient supply. This positive plant–soil–microbe cycle supports the stress resistance of secondary forests in nutrient-poor sandy habitats (Feng et al., 2022; Chen et al., 2024b).

Microbial ecological stoichiometric ratios can indicate soil nutrient limitations and vegetation nutrient requirements (Asada et al., 2022; Cui et al., 2021; Rosinger et al., 2022). In this study, the mean MBC/MBN ratio was 14.53 ± 6.08 (95% confidence interval, CI), the mean MBC/MBP ratio was 32.79 ± 16.44 (95% CI), and the mean MBN/MBP ratio was 2.28 ± 0.67 (95% CI). The mean MBC/MBN ratio was higher than both the global average of 8.1 and the Chinese average of 13.7, whereas the MBN/MBP and MBC/MBP ratios were lower than the corresponding global averages of 6.2 and 52.5 and the Chinese averages of 9.2 and 82.5 (Cleveland and Liptzin, 2007; Pan et al., 2021). These results indicate strong nitrogen and phosphorus nutrient constraints, aligning with the conclusions of a global meta-analysis of tropical forests by Camenzind et al. (2018). In both the dry and wet seasons, CA had markedly higher MBC/MBP and MBC/MBN ratios than the other forest types, whereas CO had a significantly higher MBN/MBP ratio than the other stands. The soil microbial MBC/MBN ratio has been used as an indicator of the fungal-to-bacterial ratio, with higher values suggesting a greater fungal contribution to the soil microbial community (Fauci and Dick, 1994). The elevated MBC/MBN ratio in CA may indicate a fungal-dominated microbial community, which is consistent with its identity as a common nitrogen-fixing tree species. A fungal-dominated community structure may be more favorable for nitrogen transformation and utilization (Chen et al., 2024b; Wang et al., 2010). In contrast, MBC/MBP and MBN/MBP reflect the soil capacity to supply phosphorus. Higher MBN/MBP and MBC/MBP values generally indicate insufficient available phosphorus, with microorganisms experiencing phosphorus-limited conditions (Khan et al., 2016). The relatively high MBC/MBP ratio in CA and MBN/MBP ratio in CO indicate limited soil phosphorus supply in these two stands and suggest that microorganisms are subject to substantial phosphorus limitation.

Soil microbial entropy reflects the microbial biomass supported by unit soil resources. Its variation is closely associated with microbial community composition and substrate availability (Zhang et al., 2019). As a core indicator of soil resource use efficiency and nutrient cycling intensity, microbial entropy quantifies how efficiently microorganisms convert total soil C, N, and P into living biomass. Local edaphic properties and substrate quality strongly regulate its dynamics (Anderson and Domsch, 1989; Ding et al., 2025). In this study, soil microbial entropy differed among stand types, consistent with observations from karst regions (Wu et al., 2025). Across all stands, microbial entropy consistently increased from the dry to the wet season. Improved hydrothermal conditions likely drove this increase by enhancing microbial metabolic activity and substrate use efficiency. Among stand types, higher microbial entropy in SF was associated with richer labile nutrient pools and a more stable microenvironment, which promoted microbial nutrient assimilation. In contrast, lower microbial entropy in pure plantations (CA and CO) was associated with intense nutrient leaching and poor substrate retention. These conditions constrain the conversion of total soil nutrients into microbial biomass in coastal sandy soils (Yuan et al., 2021).

### 4.3 Environmental drivers of soil microbial biomass variation in tropical coastal shelterbelts

The associations among microbial biomass stoichiometric ratios, soil microbial biomass levels, and environmental factors showed broadly similar directions in both seasons, whereas their strengths varied markedly. During the dry season, the microbial biomass level was negatively correlated with TEMP, ST, and soil phosphorus and positively associated with RH, SW, soil carbon, and soil nitrogen. In the wet season, the negative associations with TEMP and ST became stronger, whereas the negative association with soil phosphorus weakened. The positive association with TC increased, whereas strong positive associations with carbon and nitrogen were maintained. These seasonal differences were mainly linked to changes in the hydrothermal conditions. Under the low-precipitation and low-moisture conditions of the dry season, temperature and phosphorus appear to impose stronger constraints on microbial growth. In the wet season, improved hydrothermal conditions may increase the importance of carbon supply, whereas excessive precipitation may modify soil physicochemical properties and thereby change the strength of these associations. These results are consistent with those of Han et al. (2021), who reported season-dependent relationships between microbial properties and environmental factors in subtropical forests. This agrees with that of Ni et al. (2022), who demonstrated that microbial biomass in arid regions is more sensitive to moisture, whereas excessive precipitation in humid regions may inhibit microbial activity and alter the relationship between microbial biomass and precipitation. Chen et al. (2020) identified temperature and soil nitrogen as important factors associated with spatial variation in soil microbial biomass, which is also consistent with the significant associations of TEMP and soil nitrogen with the observed microbial biomass. In addition, the present results suggest that the strength of these associations is temporally heterogeneous, with the negative association with temperature intensifying from the dry to wet season, whereas the positive association with nitrogen remained stable across seasons. Overall, the environmental regulation of soil microbial biomass was expressed mainly as a seasonal adjustment in association strength rather than a fundamental shift in association direction, providing a more detailed understanding of soil microbial dynamics in seasonally dry tropical coastal shelterbelts.

The RDA results supported this trend. The correlations between microbial biomass stoichiometric ratios and soil microbial biomass with environmental factors were largely consistent between seasons, indicating that seasonal changes mainly modified the magnitude of environmental effects rather than their direction. The first two RDA axes explained more than 98% of the total variance. This relatively high explanatory power may be primarily attributed to the high homogeneity of the coastal sandy habitat and strong inherent correlations among microbial biomass-related response variables. It suggests that the selected environmental factors accounted for the variation in microbial biomass. Soil phosphorus components (TP and AP) and precipitation (PRCP) were the main explanatory variables associated with microbial characteristics. This pattern differs from those of previous studies that identified soil carbon or nitrogen as the dominant drivers of microbial biomass (Beugnon et al., 2023; Cesarz et al., 2022; Lei et al., 2025). The dominant role of P in this study is closely linked to the properties of tropical coastal sandy soils. Unlike finer-textured inland forest soils with stronger nutrient retention, coarse sandy soils have extremely low P sorption capacity. Intense seasonal rainfall drives substantial P leaching. Moreover, base-cation depletion and slightly acidic soil pH reduce available P by altering fixation and mineralization dynamics, making P availability the primary constraint on microbial growth in this habitat (Chen et al., 2019; Yuan et al., 2021). This interpretation agrees with evidence that N and P jointly regulate forest microbial biomass and that the dominant limiting nutrient varies among habitats (Liu et al., 2025). Thus, habitat context modifies the dominant environmental controls on microbial biomass.

Consistent with the core findings, exploratory supplementary analysis further indicates that the regulatory pathways of environmental factors on microbial biomass are reconfigured between seasons. This dry-season pattern is closely linked to the low water-holding capacity of coastal sandy soils. Under low rainfall, sandy soils dry rapidly, and low moisture inhibits microbial metabolism while restricting P diffusion and substrate mobility. These effects cause co-limitation by water and available P (Ni et al., 2022). In the wet season, the regulatory patterns shifted. The seasonal reversal of SW and AP effects is a distinctive feature of coastal sandy habitats. Wet-season rainfall replenishes soil moisture and relieves water limitations. Increased moisture also promotes P mineralization and substrate diffusion, allowing water and available P to promote microbial growth. However, excessive precipitation still has a direct negative effect by inducing anaerobic conditions and accelerating P leaching. This explains the persistent negative direct pathway of PRCP (Chen et al., 2019; Li et al., 2019). These findings support the close association between soil moisture and microbial biomass. They further clarify how the direction and strength of this association shift seasonally in tropical coastal shelterbelts. Environmental regulatory pathways show stronger seasonal reconfiguration in coastal sandy habitats than in inland forests with finer soils and greater nutrient retention. This pattern reflects microbial community adaptation to highly variable water and nutrient conditions in coastal ecosystems.

### 4.4 Study limitations

Several limitations should be acknowledged. This study included only three replicate plots per forest type and two seasonal sampling campaigns. Consequently, overall statistical power was relatively limited. The structural equation model, presented as supplementary exploratory analysis in the Appendix, has restricted statistical robustness due to the small sample size, and its results are only used for visualizing potential regulatory pathways rather than verifying theoretical hypotheses. The absence of microbial community composition data also limits the detailed interpretation of intrinsic microbial mechanisms. Moreover, interannual and finer-scale temporal dynamics of soil microbial biomass remain unclear. Despite these limitations, standardized field sampling and laboratory assays systematically revealed seasonal variations and key drivers of soil microbial biomass in representative tropical coastal shelterbelts. Therefore, this study provides reliable baseline data for future research. Future studies with more replicates, microbial community profiling, and long-term *in situ* monitoring should comprehensively clarify the microbial ecological mechanisms in coastal forest ecosystems.

## 5 Conclusions

This study investigated soil microbial biomass characteristics and the environmental mechanisms driving their variation across four representative tropical coastal shelterbelt forest types in Wenchang, Hainan. The results showed that the SF stands had a pronounced advantage in microbial biomass accumulation, as reflected by higher MBC, MBN, and MBP levels than those of the other forest types in both seasons. Although microbial biomass differed among the four stand types, a consistent seasonal increase throughout the testing season was observed across all types. The associations between environmental factors, stoichiometric ratios, and soil microbial biomass maintained broadly similar directions across the dry and wet seasons, whereas their strengths varied distinctly with season. In the dry season, microbial characteristics were primarily regulated by TEMP, SW, and soil phosphorus (TP and AP), whereas in the wet season, the influences of PRCP and soil carbon and nitrogen nutrients became more pronounced. Soil phosphorus and PRCP were the key factors driving changes in soil microbial biomass and its stoichiometric ratios in this region, and their dominant roles were not altered by seasonal transitions. However, the regulatory mechanisms through which environmental factors affect soil microbial biomass and its stoichiometric ratios were substantially reshaped by seasonal transitions. The strong negative regulation by PRCP, SW, and AP characterized the dry season, whereas the wet season shifted towards positive regulation by SW and AP, with the inhibitory effect of PRCP remaining relatively strong. These findings enhance the theoretical understanding of the adaptive mechanisms underlying soil microbial ecological processes in tropical coastal shelterbelts. From a management perspective, incorporating seasonal fluctuations in key influencing factors, such as soil phosphorus and PRCP, into management frameworks may help achieve a better balance between ecological protection and nutrient cycling functions, thereby providing a theoretical basis for sustainable stewardship of tropical coastal shelterbelts.

## Data Availability

The original contributions presented in the study are included in the article/**Supplementary Material**. Further inquiries can be directed to the corresponding author.
