# Supplementary material for: Soil microbial biomass in tropical coastal shelterbelts of Hainan Island: seasonal characteristics and responses to environmental factors
Source: Front Plant Sci. 2026 Aug 26;17:1925888. doi: 10.3389/fpls.2026.1925888 (PMC13553856; doi:10.3389/fpls.2026.1925888)
Supplement: Supplementary file 1 [file Presentation1.pdf]

Appendix: Structural equation modeling of environmental factors and soil microbial biomass

The following structural equation model (SEM) results are for exploratory interpretation only. Owing to the limited sample size, the statistical robustness of the models is constrained, and the results do not constitute core confirmatory conclusions of the study.

The SEM results demonstrated a clear seasonal shift in the regulation of soil microbial biomass by environmental factors between both seasons. Both models exhibited satisfactory goodness-of-fit, with Fisher's C = 3.822, P = 0.148, and AIC = 151.837 for the dry season, and Fisher's C = 0.748, $P = 0.688$, and AIC = 156.958 for the wet season. These models accounted for the direct and indirect pathways through which each environmental variable was associated with soil microbial biomass (Fig. S1).

During the dry season, environmental factors primarily exerted direct or indirect negative effects on soil microbial biomass. AP (path coefficient = −0.504, $P < 0.001$), SW (path coefficient = −0.582, $P < 0.001$), and PRCP (path coefficient = −0.852, $P < 0.001$) had highly significant direct negative effects on microbial biomass, indicating that they were the main direct factors associated with microbial biomass suppression in the dry season. The direct positive effect of TN on microbial biomass was not significant (path coefficient = 0.035, $P > 0.05$). Furthermore, PRCP strengthened the negative effects of AP (path coefficient = −0.440, $P < 0.001$) and SW (path coefficient = −0.437, $P < 0.01$) on microbial biomass by significantly suppressing these variables (Fig. S1A).

After the onset of the wet season, the direction and intensity of environmental regulation were markedly reconfigured, and its positive effects became more pronounced. SW (path coefficient = 0.664, $P < 0.01$) and AP (path coefficient = 0.745, $P < 0.01$) had significant direct positive effects on microbial biomass, making them the main positive drivers of microbial biomass during the wet season. In contrast, PRCP had a direct negative impact on microbial biomass (path coefficient = −0.876, $P < 0.01$), whereas the direct negative effect of TN was insignificant (path coefficient = −0.083, $P > 0.05$). PRCP significantly promoted AP (path coefficient = 0.611, $P < 0.001$), thereby reinforcing the positive impact of AP on microbial biomass (Fig. S1B).

These results suggest that seasonal transition substantially reconfigures the environmental regulation of soil microbial biomass. During the dry season, microbial biomass was primarily subjected to strong negative regulation by PRCP, SW, and AP. In the wet season, SW and AP were the dominant positive drivers, whereas the inhibitory effect of PRCP remained strong.

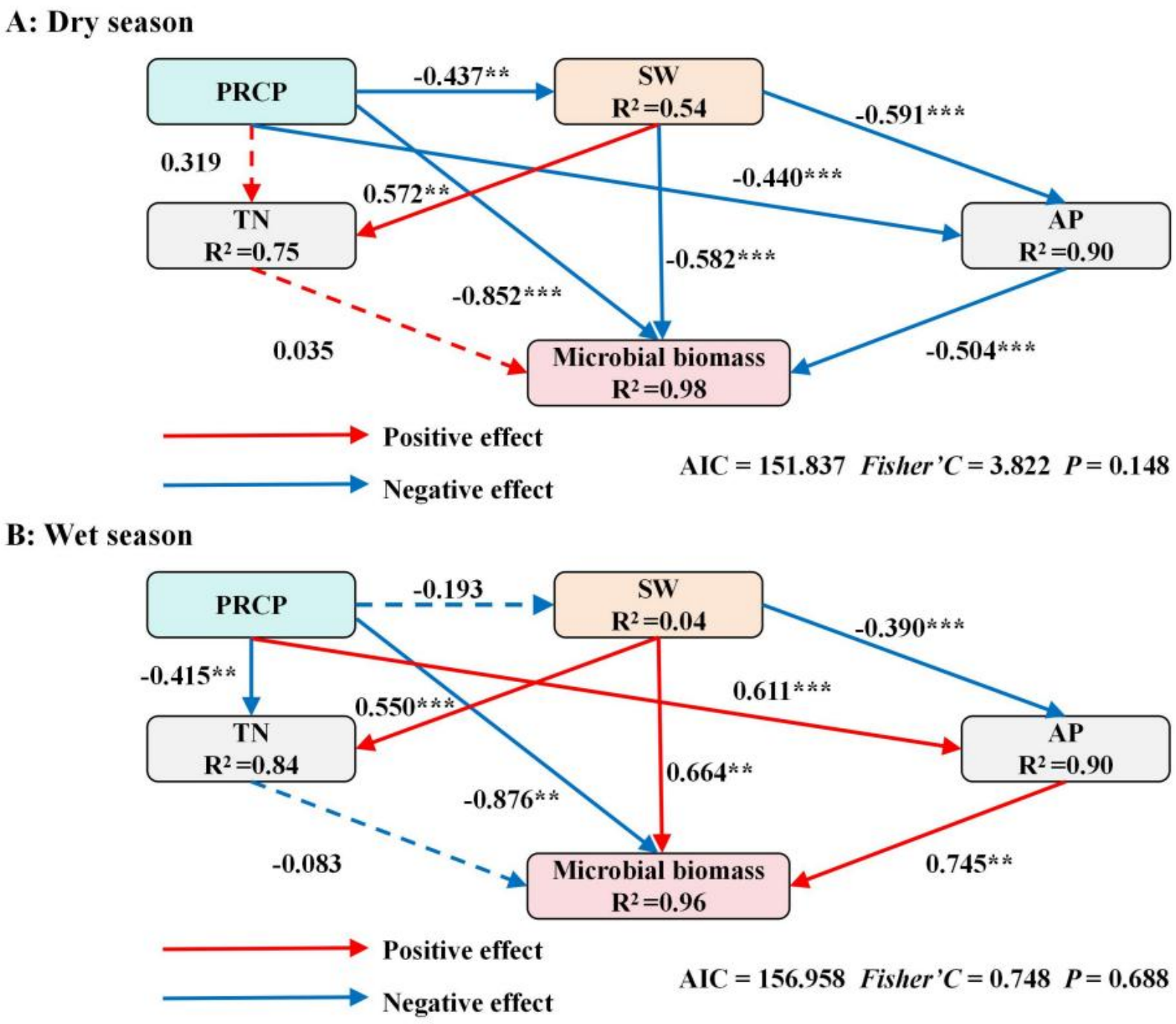

Fig. S1 Structural equation model of soil microbial biomass and its controlling factors during the dry and wet seasons. Note: *: $P < 0.05$, **: $P < 0.01$, ***: $P < 0.001$. MBP, MBN, and MBC denote soil microbial biomass phosphorus, soil microbial biomass nitrogen, and soil microbial biomass carbon, respectively. AP, AN, TP, TN, TC, pH, SW, ST, RH, TEMP, and PRCP represent soil available phosphorus content, soil available nitrogen content, soil total phosphorus content, soil total nitrogen content, soil organic carbon content, soil pH, monthly average soil water content, monthly average soil temperature, monthly average air humidity, monthly average precipitation, and monthly average air temperature, respectively.
